# Left ventricular systolic dysfunction in a patient with accidental hypothermia: a case report

**DOI:** 10.1186/1752-1947-6-429

**Published:** 2012-12-28

**Authors:** Takanao Mine, Ikuo Sato, Hideyuki Kishima, Hiroji Miyake

**Affiliations:** 1 Department of Internal Medicine, Cardiovascular Division, Hyogo College of Medicine, 1-1 Mukogawa-cho, Nishinomiya, Japan; 2 Department of Clinical Laboratory, Nishinomiya Kyoritsu Neurosurgical Hospital, 11-1 Imazu-yamanaka-cyo, Nishinomiya, Japan; 3 Department of Neurosurgery, Nishinomiya Kyoritsu Neurosurgical Hospital, 11-1 Imazu-yamanaka-cyo, Nishinomiya, Japan

## Abstract

**Introduction:**

Hypothermia is a relatively rare condition, and the effects of unintentional hypothermia on the heart are not known.

**Case presentation:**

This report describes the case of an 88-year-old Japanese man found in a coma and presenting with heart failure associated with hypothermia. He experienced sinus arrest, junctional rhythm and left ventricular systolic dysfunction with prolonged ejection time. With rewarming and treatment of his heart failure, he completely recovered over a period of two weeks.

**Conclusion:**

Prolonged ejection time might be a characteristic finding in hypothermia.

## Introduction

Differential diagnosis of a decreased level of consciousness can be difficult, as a clinical history cannot be obtained from the affected patient. A broad set of diagnostics is usually obtained, including determination of blood count, glucose, liver function and carbon monoxide levels. Brain computed tomography and a spinal tap are also often performed. Hypothermia is included within the differential diagnosis of decreased level of consciousness.

In our report we describe a case of a man with coma and heart failure associated with hypothermia. Hypothermia has been reported to reduce left ventricular systolic [1-3] and diastolic [4] function, but findings specific for hypothermia have not been described. This case involved a patient with hypothermia with a prolonged ejection time and left ventricular systolic dysfunction.

## Case presentation

An 88-year-old Japanese man presented to our emergency unit in a comatose condition with a body temperature below the lower limit of detection (<32°C when measured under the armpit; <34°C when measured at the tympanic membrane) and no external injures. He had a history of mild dementia and cerebral infarction without residual motor deficits. His medication regimen consisted of only aspirin. He was found lying in a room with a window wide open; the lowest ambient temperature recorded that night was 2°C. A physical examination showed a blood pressure of 80mmHg on palpation, a heart rate of 40 beats per minute, a respiratory rate of 10 breaths per minute, a Glasgow Coma Scale score of 4 points and an oxygen saturation (SpO_2_) of 96% on nasal oxygen at 3L/min. Warmed saline and inotropic support was administered, and our patient’s temperature normalized over three hours. His level of consciousness had normalized by the following day.

**Figure 1:**
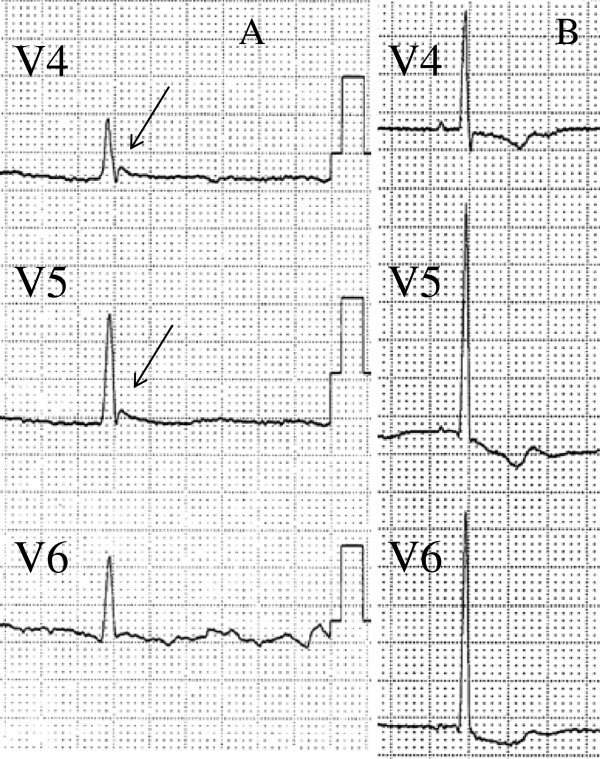
**Electrocardiogram showing progressive electrocardiographic changes.** (**A**) Osborn waves (indicated by arrow) in leads V4 to V6, on admission. (**B**) The same leads after rewarming.

**Figure 2:**
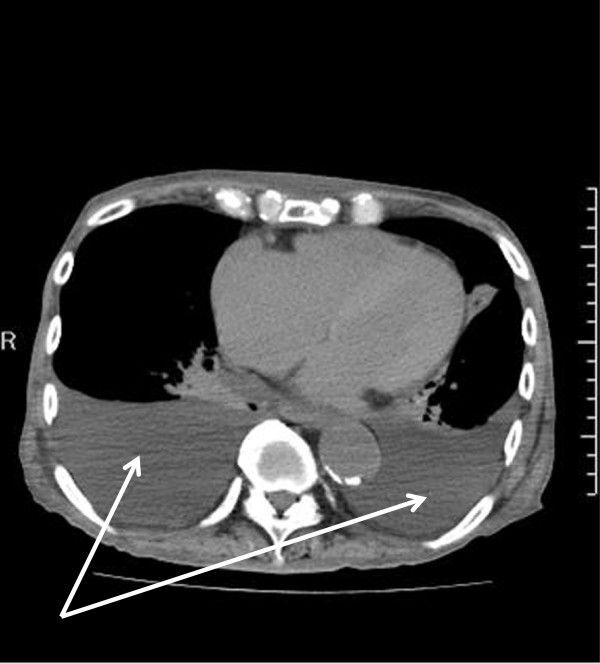
**Imaging studies of the chest.** Arrows indicate the pleural effusion.

**Figure 3:**
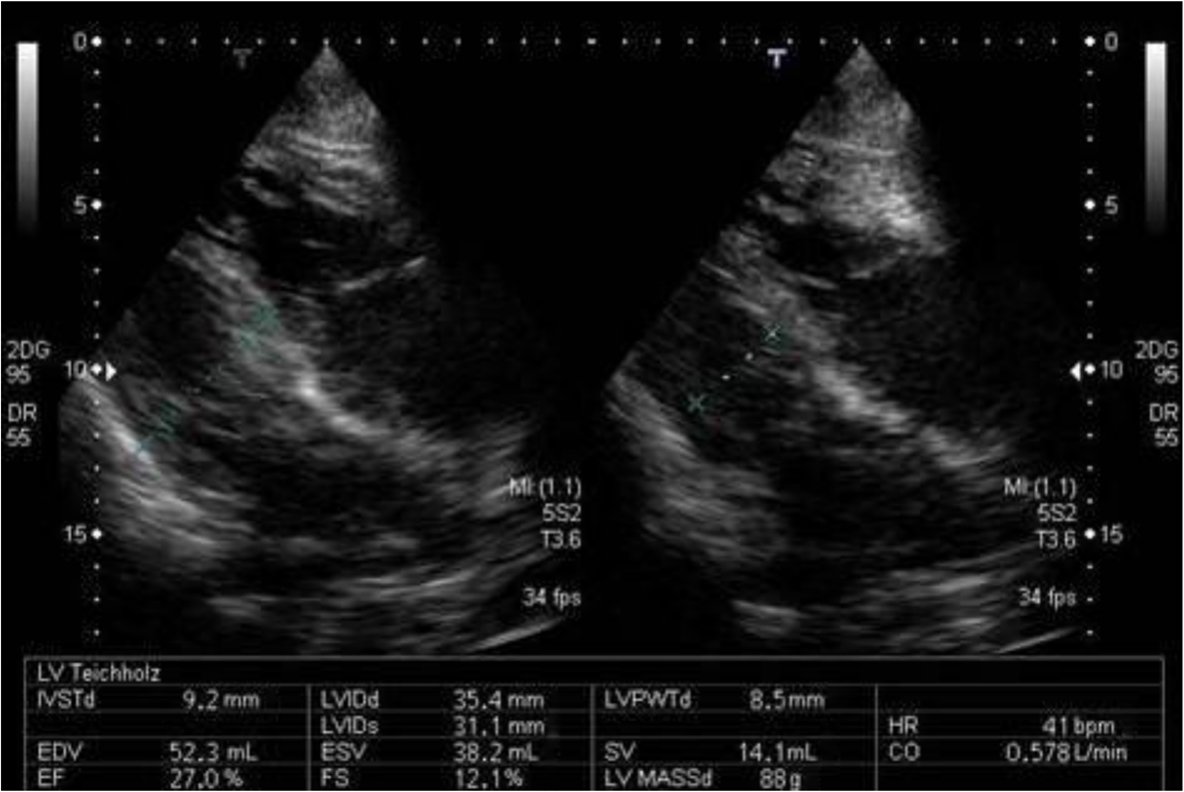
Echocardiogram of long axis view.

An electrocardiogram performed on admission showed sinus arrest and junctional rhythm with a heart rate of 40 beats per minute. Osborn waves were apparent in leads V4 to V6 (Figure 1A). Laboratory testing showed high serum creatine phosphokinase levels (448IU/L) and elevated transaminases (aspartate aminotransferase 63IU/L; alanine aminotransferase 53IU/L). Blood gas testing revealed hypercapnia (pH 7.3, partial pressure of oxygen 90.2mmHg, partial pressure of carbon dioxide 55.9mmHg, bicarbonate 27.8mmol/L, O_2 _intake 3L/min). A chest X-ray and chest computed tomography (Figure 2) showed lung congestion and pleural effusions. Echocardiography indicated left ventricular dysfunction, a left ventricular ejection fraction (LVEF) of 25%, a left ventricular end-diastolic diameter of 35mm, and an ejection time of 0.71 seconds during the preceding RR interval (2.60 seconds) (Figure 3). An additional movie file shows this in more detail (see Additional file 1). Our patient was rewarmed over three hours, and an electrocardiogram indicated sinus rhythm, a heart rate of 60 beats per minute and resolution of the Osborn waves (Figure 1B). The LVEF improved to 43% and the ejection time shortened to 0.53 seconds. After two days, the lung congestion and pleural effusions resolved. On the fifth day of rewarming, his LVEF was 45% and the ejection time reduced to 0.45 seconds.

We concluded that our patient had experienced a coma and heart failure due to accidental hypothermia. Hypothermia can impair left ventricular contraction and may specifically result in prolongation of the ejection time. His impaired cardiac function and coma resolved in response to rewarming.

## Discussion

A decreased heart rate, Osborn waves and systolic or diastolic dysfunction can be seen in the context of hypothermia [1-4], and a decreased left ventricular systolic function may persist even after rewarming [2]. However, the effects of unintentional hypothermia on the heart are unknown.

Ejection time is a hemodynamic parameter used to compute stroke volume [5] and is inversely proportional to heart rate [6]. Lance and Spodick described the relationship between heart rate and left ventricular ejection time during postural change. According to their report, the ejection time is 0.32 to 0.34 seconds when the heart rate is 40 beats per minute [6]. Teodorescu *et al*. reported that patients with heart failure and acute myocardial infarction showed significant decreases in ejection time. Moreover, ejection times are shorter than 0.25 seconds in patients with acute pulmonary edema or congestive heart failure [7]. In our case, the ejection time was clearly prolonged (0.71 seconds), which is thought to be a characteristic finding of hypothermia-induced cardiac dysfunction. While the pathophysiology of hypothermia-induced prolongation of the ejection time and systolic dysfunction is unclear, calcium+ overload due to temperature-dependent dysfunction of ion transport may play a role [8]. Aslami *et al*. showed that therapeutic mild hypothermia possibly improves oxygenation and ventilation in mechanically ventilated patients [9]. Lung congestion and pleural effusions are considered to be due to left ventricular dysfunction. The possibility of conditions such as brain disease, trauma or metabolic disorders must be excluded in the differential diagnosis of patients in coma. Our findings indicate that the prolonged ejection time observed on echocardiography may be a marker of hypothermia as the etiology of unexplained comas. Although there is still a need to conduct large-scale prospective studies, our case illustrates the characteristics of impaired cardiac function in patients with unintentional hypothermia.

## Conclusions

Our patient with hypothermia-induced coma and cardiac dysfunction presented with bradycardia, Osborn waves, systolic dysfunction and prolonged ejection time. These findings may be a characteristic finding of hypothermia-induced cardiac dysfunction. A review of additional cases is needed to determine if these signs would be helpful within the differential diagnosis of coma.

## Consent

Written informed consent was obtained from the patient’s next of kin for publication of this case report and accompanying images. A copy of the written consent is available for review by the Editor-in-Chief of this journal.

## Competing interests

The authors declare that they have no competing interests.

## Authors’ contributions

TM and IS drafted the manuscript for important intellectual content. TM, IS, HK and HM made substantial editorial revisions to the manuscript. IS made major contributions to the conception and design. All authors read and approved the final manuscript.

## Supplementary Material

Additional file 1 Videos of echocardiogram. Click here for file
